# Severe Stenosis of an Anomalous Circumflex Artery Mimicking a Chronic Total Occlusion

**DOI:** 10.1155/2018/8634275

**Published:** 2018-07-25

**Authors:** George Mawardi, Anbukarasi Maran

**Affiliations:** Coronary CTO Program, Medical University of South Carolina, 30 Courtenay Street, MSC 592, Charleston, SC 29425, USA

## Abstract

The prevalence of anomalous circumflex coronary arteries is rare. Identifying the presence of an anomalous coronary is quite easy when there is no severe stenosis. However, in the presence of severe stenosis, there is limited anterograde flow, which makes it challenging to visualize the course of the stenotic artery, and it can be assumed to be a chronic total occlusion (CTO). This case demonstrates how the anomalous circumflex artery with severe stenosis masqueraded as a CTO and the patient was treated medically for several years, despite continued symptoms. The retrograde filling of an anomalous circumflex has a specific angiographic pattern which should be recognized. This case is an excellent illustration of the said angiographic pattern.

## 1. Introduction

Coronary chronic total occlusion (CTO) can occur in up to 30% of patients with coronary artery disease. The foundation of CTO percutaneous intervention is understanding the coronary anatomy. This case report demonstrates the pitfalls of single catheter angiography in the presence of a coronary CTO. Because of the lack of understanding of the patient's coronary anatomy and failure to recognize the demonstrated specific angiographic pattern, the patient suffered with angina and did not receive appropriate treatment. Dual angiography is an essential initial step in patients undergoing CTO percutaneous coronary intervention (PCI) to define the anatomy completely. This case report illustrates how understanding the coronary anatomy with dual angiography completely changed the approach to intervention.

## 2. Case Presentation

The patient is a 65 year-old male with a past medical history significant for coronary artery disease (status post a drug-eluting stent to his right coronary artery in 2011, with occluded left circumflex), paroxysmal atrial fibrillation (status post ablation in 2015, rhythm controlled, on apixaban), abdominal aortic aneurysm (status post endovascular repair), diabetes mellitus (A1C of 6%), polymyalgia rheumatica on chronic prednisone, and colon cancer (status post resection), who was admitted to the General Medicine Service for lumbar 5-sacral 1 discitis. Cardiology was consulted for chest pain. The patient normally had stable angina pectoris but now had been experiencing unstable angina and nocturnal angina. His electrocardiogram at the time demonstrated sinus rhythm with anterolateral ST segment depressions and T wave inversions and isolated ST segment elevation in aVR. He was treated with sublingual nitroglycerin, after which the chest pain and electrocardiographic changes were resolved (EKG at rest showed sinus bradycardia, occasional premature atrial contractions (PAC), and upright T waves, with no ischemic changes).

About five months before the current episode, he had a nuclear stress test which demonstrated a small reversible perfusion defect of moderate severity in the basal, lateral region, consistent with mild ischemia. His ejection fraction was 53%.

His cardiac medications include carvedilol, aspirin, isosorbide, and lovastatin.

His physical exam was not remarkable for any physical exam findings.

His echocardiogram here showed normal ejection fraction, with inferior wall hypokinesis with a pseudonormal filling pattern.

A cardiac heart catheterization was recommended given the significant symptoms and significant electrocardiographic changes as part of his preoperative risk assessment.

The catheterization showed that the left main bifurcates into the left anterior descending (LAD) and a small left circumflex (LCx). There was also mild disease of the mid and distal left main.

**Figure 1:**
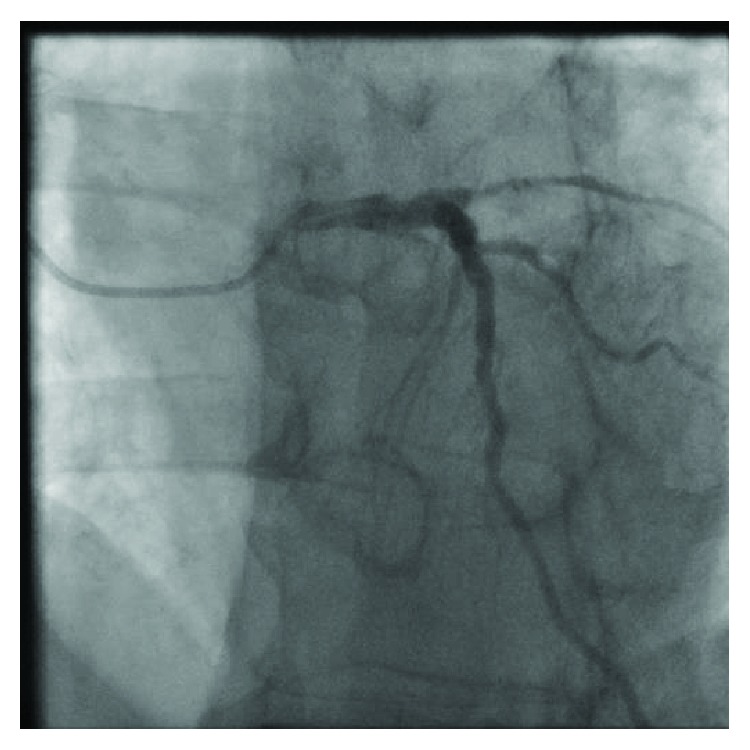
Diagnostic angiogram: left anterior oblique/cranial view demonstrating the LAD and diagonal branches.

The LAD wraps around the left ventricle apex, with diffuse mild-moderate disease. It gives off a large diagonal branch with mild diffuse disease (Figure 1).

**Figure 2:**
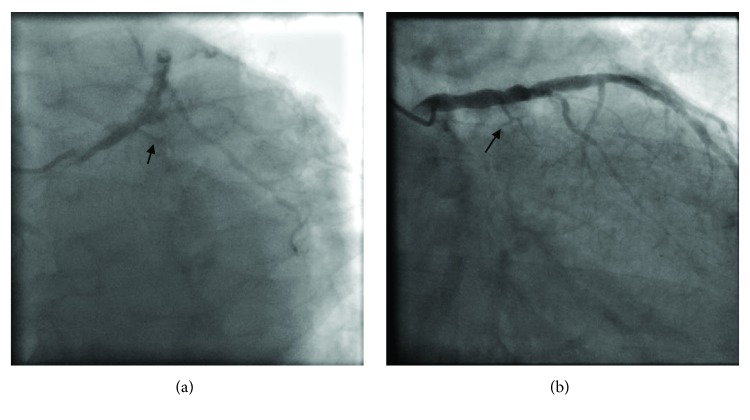
(a) Diagnostic angiogram in left anterior oblique/caudal view; (b) right anterior oblique/caudal view demonstrating the LAD ramus branch with moderate disease and diminutive circumflex branches (arrow).

The LCx was described as a small vessel with proximal occlusion at the takeoff of a large marginal branch that has mild diffuse disease (Figures 2(a) and 2(b)).

**Figure 3:**
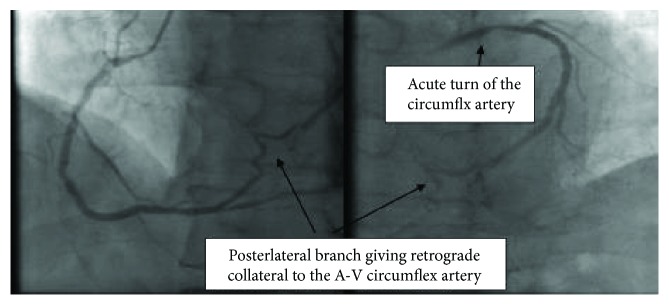
Right coronary artery with collaterals to the circumflex artery.

The right coronary artery (RCA) is a dominant vessel with a focal area of moderate eccentric disease in the mid vessel. Otherwise, it has mild diffuse disease. It gives off a large posterior descending artery (PDA) and posterolateral branch (PLB) that directly provide retrograde collateral supply to the distal atrioventricular (A-V) circumflex. The A-V circumflex turns at an acute angle away from the left main artery (Figure 3) (video file 1).

The patient underwent uncomplicated back surgery. He continued to have exertional angina (Canadian Cardiovascular Society (CCS) class III-IV) with substernal chest tightness and radiation to the shoulder with minimal activity and at rest at night. Symptoms were relieved by sublingual nitroglycerin. He was subsequently referred for hemodynamic assessment of the left main and left anterior descending with planned CTO PCI of the occluded circumflex.

**Figure 4:**
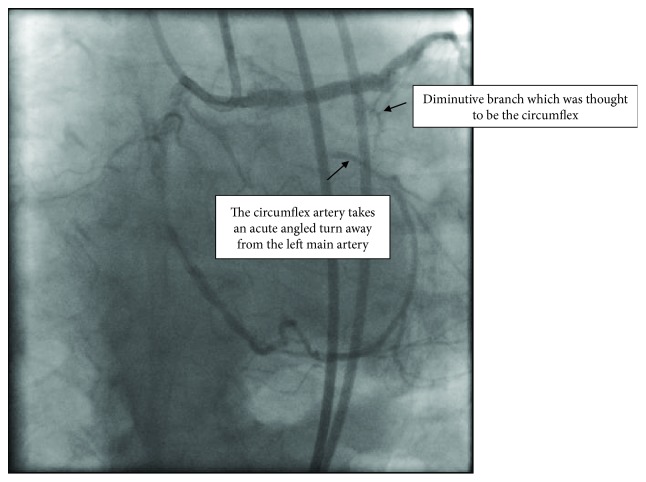
Dual angiogram demonstrating the different plane of origin of the circumflex artery.

During CTO PCI, initial dual angiogram demonstrated that the RCA collateral to the circumflex was in a different plane than the left main coronary artery (Figure 4).

**Figure 5:**
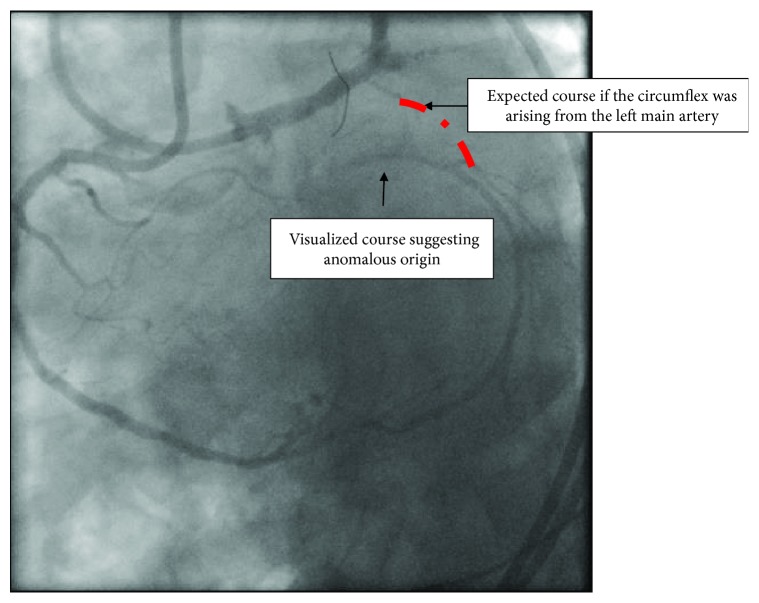
Dual angiogram demonstrating the course of the circumflex artery in a plane different from the left main artery—guide catheters in the right coronary artery and left main coronary artery in left anterior oblique/caudal view.

This raised the suspicion that the circumflex artery was probably anomalous in origin (Figure 5).

**Figure 6:**
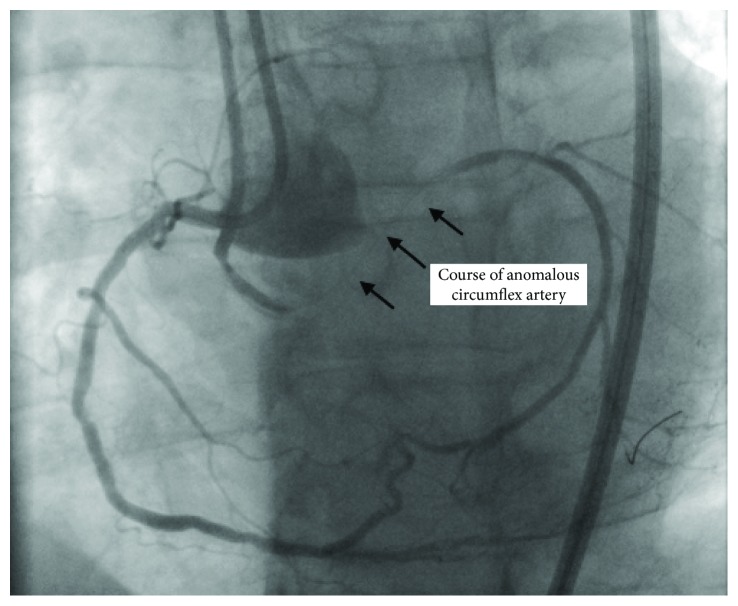
Dual angiogram with guide catheters in the right coronary artery and the anomalous origin of the circumflex artery.

Further probing of the right coronary cusp demonstrated an anomalous origin of the circumflex artery. The patient did not have CTO of the circumflex but instead had severe stenosis of his anomalous circumflex (Figure 6).

The patient was found to have an anomalous left circumflex artery, the most common coronary anomaly. Fractional flow reserve of the left main and the left anterior descending were 0.91 at rest and 0.82 at hyperaemia.

**Figure 7:**
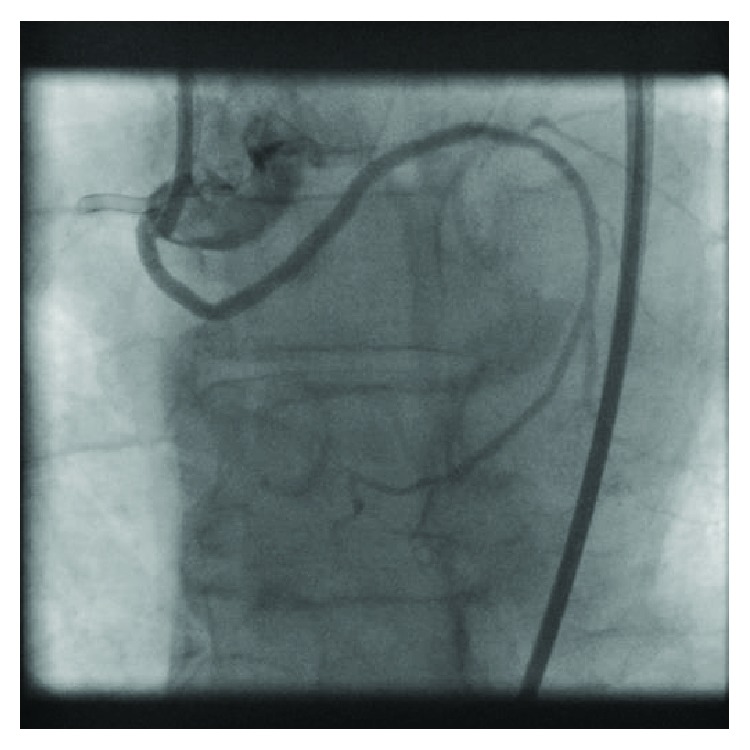
Final angiographic result after PCI of the anomalous circumflex artery.

The patient underwent successful PCI of the anomalous circumflex with drug-eluting stents (Figure 7) (video file 1).

## 3. Follow-Up

The patient did not have CTO of the left circumflex artery as described in his cardiac catheterizations previously but instead had severe stenosis of his anomalous circumflex artery. Angiographic pattern recognition of an anomalous course of a coronary artery in the presence of severe stenosis/chronic total occlusion is of importance to avoid treatment delays. The patient was followed up in a cardiology clinic 4 weeks after the procedure. He was angina free and had stopped taking long-acting nitrates.

## 4. Discussion

### 4.1. Anomalous Circumflex Artery

Coronary anomalies generally are reported to be present in about 0.2–1.3% in the population based on angiographic studies and about 0.3% based on autopsy studies [1, 2]. The most common anatomic variation is an anomalous left circumflex artery, present in about 0.7% of patients [1]. The circumflex invariably courses posterior to the aortic root. The artery can arise from a separate ostium within the right sinus or as a proximal branch of the right coronary artery. The arterial course to the aorta and pulmonary artery relationship has been associated with many serious conditions, including congestive heart failure, arrhythmias, myocardial infarction, and even sudden death [3–6]. This sudden death phenomena is thought to may be due to the fact that the vessel's tangential origin during demand can lead to ischemia and arrhythmias [7]. Retroaortic portion of the anomalous circumflex also predisposes patients to atherosclerotic disease [1]. In this case, there was severe stenosis in the anomalous circumflex artery which mimicked a CTO. Because of this, the patient was treated medically and suffered with angina for more than 6 years and did not receive appropriate treatment.

### 4.2. PCI for Chronic Total Occlusion

Even though the patient was referred for CTO PCI, he did not have a CTO. However, the systematic approach to CTO PCI helped in identifying the anomalous coronary anatomy. Chronic total occlusion interventions (CTO PCI) are increasing in frequency secondary to advances in techniques and development of the North American Hybrid Algorithm [8]. Despite the increase in CTO PCI, many non-CTO operators attempt the conventional wiring techniques, where a guide wire is attempted to be passed across the lesion without proper visualization of the distal vessel with dual angiogram. Conventional work horse wires with over-the-wire (OTW) balloons are usually used. This has been called the “Poke and Hope” technique. In some cases, this results in complete dissection and shut down of the vessel or wire perforation of the native vessel. In the case that we have described, if the “Poke and Hope” technique was to be used, it would have resulted in perforation of a small branch coming off the left anterior descending artery which was initially thought to be the diminutive left circumflex artery.

This case demonstrates the importance of dual angiography in clearly defining the coronary anatomy. Dual angiography remains the cornerstone for decision making on CTO PCI [9]. The angiogram of the right coronary artery with the right posterolateral branch giving collateral flow to the atrioventricular circumflex branch is the second most common collateral pattern for circumflex CTOs [10]; however, the turn taken by the A-V circumflex vessel is not the usual course. In the normal course of the circumflex artery, the artery runs towards the left main artery. In the case that we have described, the circumflex runs in a different plane from the left main artery, which should raise suspicion of an anomalous origin of the circumflex. Therefore, angiographic pattern recognition of this coronary angiogram is of extreme importance. If this angiographic pattern was recognized during his initial cardiac catheterization, the patient would have received appropriate treatment for his angina pectoris.

This case report serves to reiterate the unusual angiographic pattern of the anomalous circumflex artery in the presence of severe stenosis, which mimicked a chronic total occlusion. Furthermore, recognition of this angiographic pattern can facilitate further evaluation for the presence of an anomalous artery and PCI can be done in the same setting. Delays in patient care can be avoided.

## Supplementary Materials

Supplementary Materials The video starts with the diagnostic angiogram of the patient. The first image is a left anterior oblique/caudal projection demonstrating the left main artery with moderate disease in the left anterior descending (LAD) artery and moderate to severe disease in a small ramus branch. There is a small stump of the circumflex artery. The second image is a right anterior oblique/cranial projection demonstrating the LAD artery. The third image shows a left anterior oblique/cranial projection of the right coronary artery (RCA) with the posterolateral branch providing retrograde collaterals to the circumflex artery. Percutaneous intervention: the first image is a dual angiogram in the anteroposterior/caudal projection. There is a guide catheter in the right coronary artery to visualize the retrograde collateral filling and a guide catheter in the left main artery to visualize antegrade flow of the circumflex artery. This demonstrates that the collateral filling of the circumflex runs in a different plane from the circumflex stump of the left main—suggestive of an anomalous origin and course of the circumflex artery. The second image is the same dual angiogram in the left anterior oblique/caudal projection—confirming the anomalous course of the circumflex artery. The third image is a dual angiogram in the left anterior oblique projection with a guide catheter in the RCA and the second guide catheter in the anomalous ostium of the circumflex which is arising from the right coronary cusp. This demonstrates the entire course of the anomalous circumflex artery and also the severe stenosis in the proximal portion of this artery. The severe stenosis in this anomalous artery was wired with a workhorse wire and predilated with a compliant balloon. The vessel was evaluated by intravascular ultrasound which demonstrated severe fibrofatty disease in the circumflex (not visualized). The vessel was then treated with drug-eluting stents. The last image demonstrates the excellent final results with TIMI III flow in the anomalous circumflex artery.
